# Tertiary education and its association with mental health indicators and educational factors among Arctic young adults: the NAAHS cohort study

**DOI:** 10.3402/ijch.v75.32086

**Published:** 2016-09-27

**Authors:** Elisabeth Valmyr Bania, Siv Eli Kvernmo

**Affiliations:** 1 Department of Clinical Medicine, Faculty of Health Sciences, University of Tromsø – The Arctic University of Norway, Tromsø, Norway; 2 Division of Child and Adolescent Health, Department of Child and Adolescent Psychiatry, University Hospital of North Norway, Tromsø, Norway

**Keywords:** completing tertiary education, adolescents, young adults, mental health, Sami, indigenous, religion

## Abstract

**Background:**

Completed tertiary education is closely associated with employment and influences income, health and personal well-being.

**Objective:**

The purpose of the study is to explore predictors for completed tertiary education among indigenous Sami and non-indigenous young people in relation to mental health indicators and educational factors in sociocultural rural and urban contexts across the Arctic part of Norway.

**Design:**

The Norwegian Arctic Adolescent Health Study (NAAHS) is a cross-sectional, school-based survey that was conducted in 2003–2005. Of all 5,877 10th graders (aged 15–16 years) in north Norway, 83% from all 87 municipalities participated; 450 (9.2%) reported indigenous Sami ethnicity, and 304 (6.2%) reported Laestadian affiliation. Data from NAAHS were merged with registry data from the National Education Database and Norwegian Patient Register for 3,987 adolescents who gave their consent for follow-up studies.

**Results:**

Completion of upper secondary school is the only common predictor of a completed tertiary education degree for both genders. Among females, conduct problems was a significant predictor of lower level education, typically vocational professions, while among males severe mental health problems requiring treatment by the specialist health care system reduced the opportunity to complete tertiary education at intermediate and higher level. Parental higher educational level was associated with less lower education among females and less higher education among males. Men residing in the northernmost and remote areas were less likely to complete education on higher level. Males’ completion of higher level education was strongly but not significantly associated (p=0.057) with higher average marks in lower secondary school.

**Conclusions:**

The gender differences found in this study emphasize the need for gender-specific interventions to encourage, support and empower young people to attend and complete tertiary education. Young females with conduct problems choose lower or intermediate education, and males in need of specialist mental health care have half the chance to complete intermediate tertiary education compared with males not in contact with the mental health service. Closer cooperation between low threshold social services, general practitioners, mental health services and higher study institutions can help young male adults complete tertiary education.

Little research is done on circumpolar education (1), and caution is advised when comparing circumpolar education to other geographical locations because circumstances of educational access and content make comparisons difficult (1). The Nordic countries can be considered a homogeneous context within the circumpolar region. However, a knowledge gap in health status and well-being among indigenous Sami, according to the Arctic Human Development Report (AHDR) (1), is still a challenge. In general, education strongly influences standard of living, personal well-being and health (2), and this also applies to all young people in Arctic Norway (3).

Tertiary education, defined as completed university education at an intermediate or higher level, or non-university education such as a certificated vocational degree, is fundamental for employment, financial independence and good living conditions (4,5), and thus on health and well-being (6,7), and results in a more positive experience with education (6).

Tertiary education has expanded markedly in western countries over the last decades, and in 2012, tertiary qualification was achieved by every third adult in OECD countries^1^ (8). All Nordic countries within the circumpolar region are OECD members.

The minority of residents in Arctic northern Norway are Sami, the indigenous peoples of the region, along with the Kven, a national minority that originally immigrated from northern Finland and Sweden (3). Since the 1980s, a strong revitalization of culture and higher standard of living among indigenous Sami has taken place (3,9). One important factor includes a high number of well-educated indigenous Sami females, as is seen in the majority population (3). The same development is seen internationally according to OECD reports, including the Nordic countries in the circumpolar region (8). In the year 2000, adult men had higher tertiary completion rates than adult women. In 2012, however, the situation was inverted: 34% of women had completed a tertiary education compared with 31% of men (8). Historically, females in minority populations tend to have lower educational aspirations than males, possibly as a result of experiencing blocked opportunities (10). Sami males are more involved in traditional Sami occupations such as reindeer herding and fishing, in addition to the fact that those affiliated with Laestadian Christianity tend to uphold the traditional conservative male role. Laestadianism has traditionally been considered a Sami version of Lutheran Christianity, and it holds a strong religious and social position that involves conservativism and abstinence from activities considered either as sinful or not appropriate to their Christian values and beliefs, such as sexual behaviour outside marriage and substance abuse (11–14). Previous studies show that male gender and residency in sparsely populated and northernmost areas such as Finnmark County in Arctic Norway are strongly associated with lower educational aspirations (15) and a higher rate of non-completion of upper secondary school (3,16–18).

Parental socio-economic status (SES) is shown to have a great impact on educational aspirations and educational attainment in several studies (19–21), while studies in Arctic Norway have shown that parental SES has a limited effect, or no effect at all, on educational aspirations or completion of upper secondary school (15,18). Social mobility and educational equality in Norway are facilitated by national student loans for living expenses and tuition-free tertiary education (22,23), which is also the situation in other Nordic countries (1).

Mental health is found to influence educational attainment (24–27).

Females seem to complete school despite experiencing mental health symptoms such as emotional problems (28,29). Studies have shown significantly higher frequency of females seeking help from school health services and general practitioners, but an equal proportion of females and males sought help from specialist health care services (30). Aside from this, social problems among females are associated with female drop-out from upper secondary school (18). Males with externalized symptoms such as hyperactivity symptoms and conduct problems tend to have higher drop-out from upper secondary school (28,29) as well as lower attendance rates in tertiary education (27) than peers without these problems.

Students’ average mark is the single most prominent finding from several studies of completion of upper secondary school (31), which is shown to have great impact on completing tertiary education (31). In Arctic Norwegian students, higher educational aspirations are associated with higher marks, and lower average marks with a lower level of educational aspirations (15).

In recent decades, several studies have shown that the mental health of Sami youth is as good as that of non-Sami counterparts (30,32–35), and studies have shown that the overall frequency of help seeking was similar between Sami and non-Sami adolescents (30). Sami youth report less substance abuse than their non-indigenous peers (13,30,32–35), possibly related to the influence of Laestadianism and of its strong abstinence norm (13).

No previous studies have explored how mental health indicators in adolescence and young adulthood influence the completion of tertiary education among Arctic indigenous Sami and non-indigenous young people, when controlling for self-reported educational aspirations and upper secondary school history. The first aim of this study was to explore completed tertiary education at university level as well as an accredited tertiary vocational level in a large population of young people in Arctic Norway. The second aim was to determine the importance of predictors such as gender, ethnicity, residency, religious affiliation, mental health and educational issues.

We expected that poorer family finances, male gender combined with Sami ethnicity and living in sparsely populated areas, and mental health symptoms, such as self-reported social problems and conduct problems, as well as mental health problems requiring specialist mental health care would be factors increasing the risk of not completing tertiary education, whereas positive educational factors such as higher parental education, self-reported educational aspirations, completion of upper secondary school and high average mark, as well as Laestadian religious affiliation, would promote tertiary education.

## Methods

### Sample and procedure

The Norwegian Arctic Adolescent Health Study (NAAHS) was conducted from January 2003 until January 2005. All 10th grade students in all lower secondary schools in the three northernmost Norwegian counties were invited to participate in this study. The participants included 4,881 of 5,877 adolescents who were in the 10th grade in lower secondary school, and they were either 15 or 16 years old. The following response rates were observed for the total sample and samples for the three counties: totally 83%, Finnmark 71%, Troms 82% and Nordland 88%, respectively. The participating sample consists of all 10th graders who were present at school when the survey was conducted, except for students at one school who refused to participate.

The questionnaires were administered during a 2-hour period in a classroom setting monitored by project staff, and non-attending students completed them later. The questionnaire was available in both the Sami and Norwegian languages.

The adolescents had to provide written consent for later follow-up studies including linkage to registry data. The parents of these adolescents were given written information about the study. In total, 3,987 (68%) of the adolescents gave their consent to use registry data. To achieve this, we linked the NAAHS, with the Norwegian Patient Register (NPR) and the National Education Data Base (NUDB), which together provide information about each person's contact with the specialist health care system, completion of upper secondary school and different levels of completed tertiary education on university level as well as certificated vocational education. This allowed us to follow each person's educational progress up until their early 20s.

The study was approved by the Regional Committee for Medical and Health Research Ethics.

### Measures

#### Outcome variable from NUDB

*Completed tertiary education* is defined *as* “Higher educational level” (university, 5 years and longer) (1), “Intermediate educational level” (university, 3–5 years) (2), “Lower educational level” (vocational level) (3) and “Not-completed tertiary education” (other) (4). The four categories are not ordinal, but represent four different levels. Not-completed tertiary education is the reference group.

#### Explanatory variables from the NAAHS study, NUDB and NPR

*Gender*: Female gender was used as the reference group.

*Residency* refers to the county where the adolescent lived during lower secondary school. The three northernmost counties in Norway were compared: Nordland, Troms and Finnmark of which Finnmark County is the northernmost, most remote and sparsely populated. Nordland County, the southernmost, has the largest number of inhabitants and is used as the reference group.

*Sami ethnicity* was measured by an assessment of parents’ ethnicity, Sami language competence in parents, grandparents and the participants, and ethnic self-identification. Participants who had one or more of these affiliations present were classified as having Sami ethnicity (36). Non-Sami ethnicity was the reference group.

*Laestadian affiliation* was measured by the youth's reports on their own, the parents’ or the grandparents’ affiliation to the Laestadian religious movement. Participants having one or more of the affiliations were classified as having Laestadian affiliation. Non-Laestadian affiliation was the reference group.

*Parental educational level:* Parents’ education, registered when the participants were 15–16 years, was used. Parents’ highest accomplished year of education was obtained from Statistics Norway's register on education. In the analyses, parents’ education was categorized as “Higher educational level” (university, 5 years and longer) (1), “Intermediate educational level” (university, 3–5 years) (2), “Lower educational level/upper secondary school (vocational level)” (3) and “Lower secondary school” (4). Parental higher educational level (1) was the reference group.

*Family financial situation* was measured by the adolescents’ self-report and categorized as: “Poor” (1), “Average” (2), “Good” (3) and “Very good” (4). Family financial situation being poor (1) was the reference group.

*Mental health*: *Strength and Difficulties Questionnaire* (SDQ) (37) consists of five subscales which adolescents answered in the school survey in lower secondary school. Three subscales were used: the Emotional Symptoms Scale (SDQ-emotions) (α=0.70), the Hyperactivity Scale (SDQ-hyper) (α=0.64) and the Conduct Problems Scale (SDQ-conduct) (α=0.47). The subscales have five items each with scores from 0 to 2 on each item, indicating: 0=not correct, 1=correct sometimes and 2=totally correct. The total score for these subscales ranged from 0 to 10, with the lowest score indicating the least amount of difficulty. Each question scored from 0 to 2, with 0 indicating no problems and 2 indicating great worries and large problems. The scales were operationalized on the basis of the mean scores of the five questions.

*
Contact with specialist mental health service* is measured by data from the NPR on the use of mental health services after the age of 18. No-contact is coded=0, and yes, contact either/or both in-patient and out-patient is coded=1. No-contact with specialist mental health service was the reference group.

*Average marks are* based on the four major subjects: mathematics, Norwegian, English and social sciences in lower secondary school. The Norwegian system of school marks ranges from 1 to 6 (1–2=poor, 3=average, 4=good, 5=very good and 6=excellent). For this variable to be included in the analyses, a reported mark in at least three out of the four subjects must be present.

*Educational aspirations* were measured by the question, “What is the highest educational level you have planned” from the cross-sectional survey NAAHS, completed during 2003–2005. The students could only respond to one option: “University or university college on high level” (lector, solicitor, civil engineer, dentist, doctor, psychologists, civil economist) (1), “University or university college on middle level” [Norwegian university degree (3.5–4.5 years), teacher, social worker, nurse, police, engineer, journalist] (2), “High school diploma level” (3), “Vocational education on upper secondary school level” (chef, hairdresser, builder, electrician, assistant in health and social care, etc.) (4), “One year in high school” (5), “Other: open spot to fill in by pen” (6), or “I have not decided” (g).

The options were recoded into four categories: higher level (1), intermediate level (2), lower level (3) and undecided (4) (undetermined on the choice of profession). The undecided category was the reference group.

*Completion of upper secondary school* is defined as achieving a complete upper secondary school diploma within 5 years after completing lower secondary school. Not having completed upper secondary school within 5 years after lower secondary school was defined as “non-completion”. Completion was the reference group.

### Statistical analyses

Groups were compared using Pearson's chi-squared test for categorical data, and Student's t-test and one-way ANOVA for continuous data. Multinomial logistic regression analysis was carried out with completed tertiary education as the dependent variable, unadjusted and fully adjusted, stratified for females and males. In the gender-stratified unadjusted analyses, mental health and educational factors were controlled for sociodemographic variables; ethnicity, religious affiliation, residency, parental educational level and family financial situation. The fully adjusted analyses are based on all covariates that were significant or showed an association from the unadjusted analyses.

The p-values ≤0.05 were considered statistically significant. The statistical package SPSS 21 was used for all analyses.

## Results

Of the respondents in the registry data sample (N=3,987), 50.1% were females and 49.9% were males. About 10% of the respondents from the registry data sample reported Sami ethnicity, and 20.5% of Sami youngsters reported Laestadian affiliation, while 5.4% in the non-Sami population reported Laestadian affiliation. Most Sami lived in the northernmost Finnmark County (29.8%), 10% in Troms County and 5% in Nordland County, while the remaining inhabitants in the counties are non-Sami.

Gender-stratified analysis was applied due to the gender differences in the following independent mental health factors: SDQ-emotional, SDQ-conduct and SDQ-hyperactivity and the educational factors – average marks and educational aspirations (Tables I and II).

**Table I:** Mental health indicators of the sample by registry data Norwegian Patient Register (NPR) and National Arctic Adolescence Health Survey (NAAHS) variables (10th grade)

		Gender (N=3,987)						Ethnicity (N=3,987)				
				
	Total N (MV)	Females		Males		χ^2^/p	Total N (MV)	Sami		Non-Sami		χ^2^/p
Mental health specialist service	3,987	N	%	N	%	1.634^p=0.109^	3,645 (342)	N	%	N	%	0.367^p=0.304^
No-contact	3,444	1,706	85.7	1,738	87.1		3,148	319	87.4	2,829	86.3	
Contact	543	285	14.3	258	12.9		497	46	12.6	451	13.8	
		Mean	SD	Mean	SD	t/p		Mean	SD	Mean	SD	t/p
SDQ-emotional	3,944 (43)	3.453	2.262	1.691	1.728	27.482^p<0.001^	3,643 (344)	2.517	2.047	2.577	2.222	0.493^p=0.621^
SDQ-hyperactivity	3,943 (44)	4.283	2.153	4.051	2.128	3.408^p<0.001^	3,642 (345)	4.008	2.258	4.164	2.130	1.319^p=0.187^
SDQ-conduct	3,946 (41)	1.995	1.417	2.281	1.675	5.778^p<0.001^	3,643 (344)	2.442	1.747	2.084	1.531	4.175^p<0.001^

MV, missing values; SDQ, Strength and Difficulties Questionnaire.

**Table II:** Educational factors and achievement indicators of the sample by registry data National Education Data Base (NUDB), based on National Arctic Adolescence Health Survey (NAAHS) variables (10th grade)

		Gender (N=3,987)						Ethnicity (N=3,987)				
				
		Females		Males				Sami		Non-Sami		
								
	Total N (MV)	Mean	SD	Mean	SD	χ^2^/p	Total N (MV)	Mean	SD	Mean	SD	χ^2^/p
Average marks	3,541 (446)	4.098	0.780	3.802	0.783	11.291^p<0.001^	3,264 (723)	3.929	0.786	3.969	0.785	0.866^p=0.387^
Educational aspirations	3,928 (59)	N	%	N	%	99.918^p<0.001^	3,605 (382)	N	%	N	%	6.453^p=0.092^
Higher level	865	472	24.0	393	20.1		812	86	24.0	726	22.4	
Intermediate level	722	453	23.0	269	13.7		664	59	16.4	605	18.6	
Lower level	1,224	494	25.1	730	37.2		1,102	95	26.5	1,007	31.0	
Undecided	1,117	549	27.9	568	29.0		1,027	119	33.1	908	28.0	
Upper secondary school	3,981 (6)					0.062^p=0.414^	3,639 (348)					2.842^p=0.052^
Completion	2,518	1,253	63.1	1,265	63.4		2,283	213	58.7	2,070	63.2	
Non-completion	1,463	734	36.9	729	36.6		1,356	150	41.3	1,206	36.8	
Completed tertiary education	3,987					5.331^p=0.149^	3,645 (342)					2.892^p=0.408^
Higher level	217	111	5.6	106	5.3		200	26	7.1	174	5.3	
Intermediate level	733	371	18.6	362	18.1		664	67	18.4	597	18.2	
Lower level	994	465	23.4	529	26.5		906	95	26.0	811	24.7	
Not-completed tertiary education	2,043	1,044	52.4	999	50.1		1,875	177	48.5	1,698	51.8	

MV, missing values.

Completed tertiary education was fairly equally and non-significantly distributed between genders, with a prevalence of approximately 5% for higher level and approximately 18% for intermediate level. About one-quarter of the young people completed lower level (non-university tertiary education), while more than half of the young people had not yet completed any tertiary education. Sami young people had somewhat but not significantly higher rates of higher education completion (7.1%) than non-Sami (5.3%) and lower rates of not-completed tertiary education (48.5%) than non-Sami (51.8%) (Table III). Females who completed lower level were shown to have more conduct problems (SDQ-conduct) (F=4.383, p=0.004).

**Table III:** Sociocultural characteristics of the sample by registry data National Education Data Base (NUDB), based on National Arctic Adolescence Health Survey (NAAHS) variables (10th grade)

	Total N (MV)	Gender (N=3,987)					Total N (MV)	Ethnicity (N=3,987)				
		
		Females		Males		χ^2^/p		Sami		Non-Sami		χ^2^/p
Ethnicity/gender	3,645 (342)	N	%	N	%	0.145^p=0.372^		N	%	N	%	
Sámi	365	187	10.2	178	9.8							
Non-Sámi	3,280	1,646	89.8	1,634	90.2							
Religious group	3,978 (9)					12.117^p<0.001^	3,645 (342)					116.267^p<0.001^
Laestadian	254	100	5.0	154	7.7		253	75	20.5	178	5.4	
Non-Laestadian	3,724	1,882	95.0	1,842	92.3		3,392	290	79.5	3,102	94.6	
Residency	3,987					1.826^p=0.401^	3,645 (342)					290.663^p<0.001^
Nordland County	2,104	1,033	51.9	1,071	53.6		1,923	89	24.4	1,834	56.0	
Troms County	1,310	674	33.9	636	31.9		1,195	119	32.6	1,076	32.8	
Finnmark County	573	284	14.2	289	14.5		527	157	43.0	370	11.2	
Parental educational level	3,974 (13)					1.471^p=0.689^	3,633 (354)					1.169^p=0.760^
Higher educational level	337	167	8.4	170	8.5		305	29	8.0	276	8.4	
Intermediate educational level	1,285	633	31.9	652	32.8		1,176	110	30.2	1,066	32.6	
Lower educational level/upper secondary school	1,892	963	48.5	929	46.7		1,729	182	50.0	1,547	47.3	
Lower secondary school	460	222	11.2	238	12.0		423	43	11.8	380	11.6	
Family financial situation	3,918 (69)					0.326^p=0.955^	3,584 (403)					4.050^p=0.256^
Poor	136	66	3.4	70	3.6		129	10	2.8	119	3.7	
Average	1,355	682	34.9	673	34.3		1,250	116	32.3	1,134	35.2	
Good	2,129	1,057	54.0	1,072	54.6		1,933	198	55.2	1,735	53.8	
Very good	298	151	7.7	147	7.5		272	35	9.7	237	7.3	

MV, missing values.

Other predictors such as SDQ-emotions, SDQ-hyperactivity and average mark were not significant (data not shown).

Unadjusted logistic regression analyses when controlled for sociodemographic variables showed that Laestadian affiliation in females was associated with a lower level of completed tertiary education. More self-reported emotional symptoms (SDQ-emotions) and conduct problems (SDQ-conduct) predicted completion of tertiary lower educational level for females when adjusted for sociodemographic variables (Table IV). Parental intermediate educational level was found to be negatively associated with male intermediate education level. Univariate logistic analysis adjusted for sociodemographic variables showed that mental health symptoms by SDQ-emotions were significant for completion of higher level tertiary education among males, and average mark for intermediate level. A strong but not significant association was found between males’ educational aspirations for intermediate level and completed tertiary education on intermediate level (p=0.056).

**Table IV:** Logistic regression analysis with completed tertiary education as a dependent variable [adjusted for sociodemographic factors (OR, 95% CI)]

	Completed tertiary education^a^					
	
	Higher level		Intermediate level		Lower level	
			
Covariate	Females	Males	Females	Males	Females	Males
Sociocultural factors						
Sámi ethnicity						
Laestadian affiliation					1.820 (1.006–3.292)^p=0.048^	
Troms County^b^						
Finnmark County^b^						
Parental intermediate educational level^c^				0.507 (0.281–0.915)^p=0.024^	0.472 (0.283–0.786)^p=0.004^	
Parental lower (vocational) educational level^c^					0.562 (0.376–0.839)^p=0.005^	
Parental lower secondary school, educational level^c^					0.497 (0.326–0.757)^p=0.001^	
Family finances: average^d^						
Family finances: good^d^						
Family finances: very good^d^						
Mental health indicators						
Contact with specialist mental health service						
SDQ-emotions		1.121 (1.004–1.252)^p=0.043^			1.055 (1.002–1.110)^p=0.041^	
SDQ-hyperactivity						
SDQ-conduct					1.137 (1.048–1.233)^p=0.002^	
Educational factors						
Non-completion upper secondary school	0.007 (0.001–0.048)^p=0.001^	6.616E-11^p=0.001^	0.051 (0.032–0.079)^p=0.001^	0.071 (0.048–0.107)^p=0.001^	0.180 (0.138–0.237)^p=0.001^	0.251 (0.196–0.323)^p=0.001^
Average mark				1.209 (1.015–1.442)^p=0.034^		
Educational aspiration: lower level^e^						
Educational aspiration: intermediate level^e^						
Educational aspiration: higher level^e^						

SDQ, Strengths and Difficulties Questionnaire. Reference groups:

a Not completed tertiary education.

b Nordland County.

c Parental educational level – higher.

d Family financial situation – poor.

e Educational aspiration undecided.

The fully adjusted logistic regression analyses showed that non-completion of upper secondary school is significantly associated with not attending tertiary education on all levels for both females and males, and strongest among males (Table V). Males residing in Finnmark County showed a significantly lower rate of completion of higher level education (Table V). Parental education showed a significant impact for males on completed higher level education, and females on lower level (Table V). Mental health factors such as higher SDQ-conduct problems were significantly related to lower level education for females, while the effect of a higher score of SDQ-emotion symptoms on higher level education showed a strong, but not significant association among males (p=0.062) in the fully adjusted analyses. The impact of contact with the mental health specialist service was highly significant for males’ completion of education on intermediate level, while higher average mark was strongly but not significantly associated with higher level education (p=0.057).

**Table V:** Logistic regression analysis with completed tertiary education as a dependent variable [fully adjusted model (OR, 95% CI)]

	Completed tertiary education^a^					
	
	Higher level		Intermediate level		Lower level	
			
	Females	Males	Females	Males	Females	Males
Sociocultural factors						
Laestadian affiliation						
Troms County^b^						
Finnmark County^b^		0.460 (0.246–0.859)^p=0.015^				
Parental intermediate educational level^c^		0.307 (0.118–0.801)^p=0.016^		0.442 (0.227–0.861)^p=0.016^	0.474 (0.275–0.818)^p=0.007^	
Parental lower (vocational) educational level^c^		0.405 (0.198–0.828)^p=0.013^			0.501 (0.322–0.778)^p=0.002^	
Parental lower secondary school, educational level^c^		0.380 (0.178–0.813)^p=0.013^			0.489 (0.309–0.773)^p=0.002^	
Mental health indicators						
Contact with specialist mental health service				0.551 (0.368–0.826)^p=0.004^		
SDQ-emotions						
SDQ-hyperactivity						
SDQ-conduct					1.146 (1.042–0.246)^p=0.005^	
Educational factors						
Non-completion upper secondary school	0.014 (0.003–0.057)^p=0.001^	6.916E-11^d^^,p=0.001^	0.069 (0.046–0.103)^p=0.001^	0.067 (0.051–0.089)^p=0.001^	0.187 (0.142–0.246)^p=0.001^	0.219 (0.184–0.261)^p=0.001^
Average mark						

SDQ, Strengths and Difficulties Questionnaire. Reference groups:

a Not completed tertiary education.

b Nordland County.

c Parental educational level – higher.

d Very low numbers, both OR and CI.

For both gender and regardless of ethnicity, educational aspirations showed no impact for any level of completed tertiary education.

## Discussion

A linkage of cross-sectional data with registry data addressing mental health problems by NPR and educational factors from lower secondary school and onwards to tertiary level by NUDB has not been done previously, to our knowledge.

In this study, completing upper secondary school is the most prominent finding and significant factor associated with completed tertiary education. Since 2005, Norway, like France, has legislated recognition of work-related and informal competence for a maximum period of 5 years, and this can be acquired until the young adult is 25 years old, called the 25/5 rule, counting towards admission to tertiary education. The legal recognition is shown to be implemented in practice in this study, as 23.1% of the young adults who did not complete upper secondary school still complete certified vocational level education, presented as non-university tertiary education (5). More than 8% of young people who dropped out of upper secondary school nevertheless got a university degree at intermediate level, typically a bachelor's degree. However, completion of higher level education was generally low in this study, and rare among young people who had not completed upper secondary school. The findings prove that social mobility is possible through tertiary education, including among young adults who dropped out of upper secondary school. Nevertheless, a larger worry is that 21.8% of young adults who had completed upper secondary school did not complete tertiary education on any level at this age. Although the connection between completed upper secondary school and completed tertiary education is highly significant for the age group examined, there is a possibility for later completion of tertiary education. One possibility is gaining informal and non-formal competence through work experience and reaching the age of 25. Another factor is that some adolescents might drop-out of upper secondary school and later manage to retake the subjects they lack, and thereby qualify on regular premises to attend tertiary education.

As hypothesized for males, residency in Finnmark County is significantly associated with less higher level education. Previous studies have shown the same results for non-completion of upper secondary school for the same group of young people (3,16–18).

In other studies, education and SES are found to be highly correlated (1,20,21). In this study, only parents’ higher level of education was found to be significant for females’ completion of lower level education and males’ higher level education. Young males did not seem to be influenced by their parents’ higher educational level for completion of higher tertiary education in the same way as for the females. Young females with highly educated parents do not choose tertiary education on lower level. One interesting finding in a socio-economic perspective is that family finances are not found to have any significant impact on completion of tertiary education in this study. Previous studies have shown the same lack of association for educational aspirations and parental SES by work (15). In this study, socio-economic factors such as family finances showed little impact on completed tertiary education. However, the last socio-economic factor examined, parental educational level, was highly significant among females’ and males’ completion of tertiary education, as found in other studies (38,39). In egalitarian Norway, as well as other Nordic countries, finances might be less important, as higher education is tuition free and nationally funded student loans are available. Nevertheless, parental higher education can lead to engagement, support and expectations by the parents who act as educational role models for the youngsters (25,26).

Mental health factors such as more self-reported adolescent conduct problems influenced females’ completion of lower level significantly, and we found a strong association in the same direction at the intermediate level, typically the bachelor's degree. More conduct problems can be associated with concentration difficulties and impulsiveness (28,29). Young adults with higher scores in the area of conduct problems can consciously limit the number of study years as a way to accommodate their challenges, or choose a vocational education that is less cognitively demanding. Internalizing symptoms such as higher score on adolescent emotional symptoms are protective for males’ higher educational level, as seen in other studies concerning females’ completion of upper secondary school (18,28,29). However, when there are more serious mental health problems in young adulthood that need treatment in the specialist mental health service, males have a significantly lower chance of completing higher and intermediate level education, as found in other studies (27). The finding can be connected to less service-seeking among males in the primary health care services, typically school health services and general practitioners (30), which could help prevent serious mental health problems.

### Strengths and limitations

The major strength of this study is the population-based design with a high response rate adding to the generalizability of the study, by its valid and reliable data. Data from the cross-sectional study are merged to high-quality registry data.

Reliability and validity of brief scales, as SDQ, may be questioned (40–42). Cronbach's alpha was applied as a measure of internal consistency reliability, with a value of 0.70 or more considered reliable. The Conduct Problems Scale (SDQ-conduct) had a lower value, while subscales such as the Emotional Symptoms Scale (SDQ-emotions) and the Hyperactivity Scale (SDQ-hyper) could be considered reliable.

The NAAHS data were collected 6–10 years before the outcome data in NUDB and NPR, which limited the possibility of examining the impact of the predictors in late lower secondary school through completion of tertiary education. Future surveys should use a longitudinal follow-up design to examine the impact of the predictors during the whole education trajectory.

The NAAHS survey was conducted during school hours, and in a classroom setting. The physical setting may have affected the response due to selection bias.

The NUDB includes information and outcomes pertaining to all aspects of education, from primary school to higher education. The outcome variable is therefore considered reliable.

## Conclusion

Tertiary education is strongly associated with completion of upper secondary. Still, this study shows that even young adults with non-completion of upper secondary school completed lower and intermediate tertiary education. Young adults who dropped out of upper secondary school have access to higher education by the legislated 25/5 rule, which gives young adults entitlements and credits for age and relevant work practice. On the other hand, a substantial part of the respondents with a completed upper secondary school did not complete a degree in tertiary education. This can be explained by the relatively young age of the studied population (23–25 years old), who postpone tertiary education because of employment or other activities. Norwegian students have the possibility to attend tuition-free tertiary education and receive student loans and grants at any age.

The gender differences found in this study should be emphasized. Young adults with various mental health problems need support and low threshold services, both from the educational institutions and from general practitioners and social and healthcare services to enable females and males to complete higher education. The results from this study suggest that young males from the remote north in particular can be empowered, encouraged and supported by systematic follow-up to enter and complete tertiary education.

## Footnotes

1 The Organization for Economic Co-operation and Development, which is a group of western and market-capitalized countries stating they are working for a better life and better policies.
